# Supplementary material for: Precocious Downregulation of Krüppel-Homolog 1 in the Migratory Locust, Locusta migratoria, Gives Rise to An Adultoid Phenotype with Accelerated Ovarian Development but Disturbed Mating and Oviposition
Source: Int J Mol Sci. 2020 Aug 22;21(17):6058. doi: 10.3390/ijms21176058 (PMC7503607; doi:10.3390/ijms21176058)
Supplement: Supplementary file 1 [file ijms-21-06058-s001.pdf]

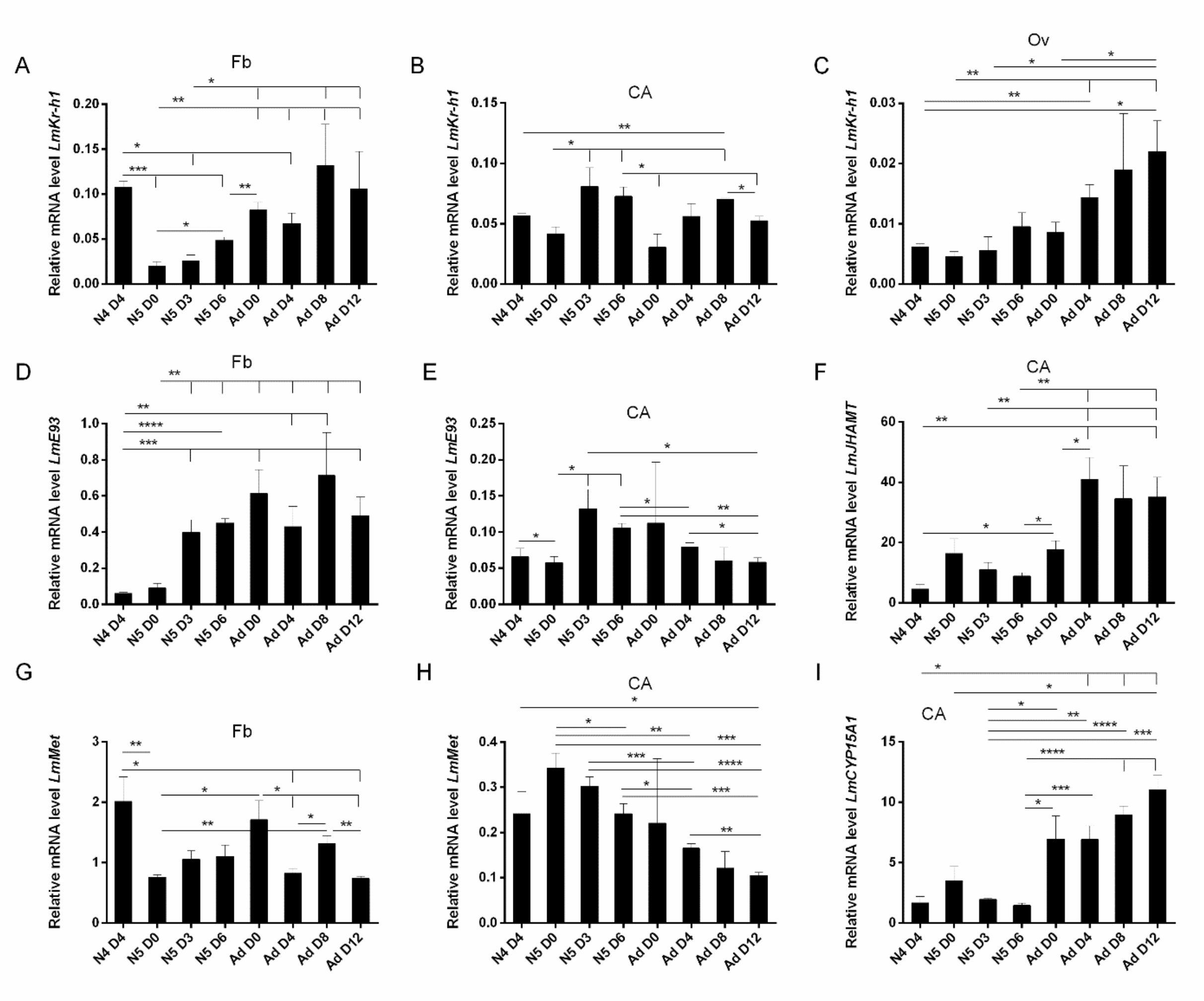

Figure S1. Transcript profiles of MEKRE93 and JH biosynthesis pathway components in control (*dsGFP* injected) female locusts during the last nymphal stages and the first gonadotrophic cycle. Relative levels of *LmKr-h1* (A + B), *LmE93* (D + E) and *LmMet* (G + H) transcripts were determined in the fat body (A + D + G) and the CA (B + E + H) of control (*dsGFP*) females starting from day 4 in the fourth nymphal stage (N4 D4) until day 12 in the adult stage (Ad D12). Relative levels of *LmKr-h1* transcripts were also measured in the ovaries (C). Relative quantities of *LmJHAMT* (F) and *LmCYP15A1* (I) transcripts were measured in the CA. The data represent mean ± S.E.M. of four independent pools of three animals, run in duplicate and normalized to *rps13* + *CG13220*, *CG13220* + *TubA1* and *rp49* + *rps13* transcript levels for fat body, ovary and CA samples, respectively. Statistically significant differences between the measurements were found via a t-test on log-transformed data (with or without two-sided Welch's correction) and are indicated by (an) asterisk(s) (* $p < 0.05$; ** $p < 0.01$; *** $p < 0.001$; **** $p < 0.0001$).

ATGGTGGGCTACTTCAACGGCGAGGGMCAGGCGCAGGGYCCCGGCCCGGCGCCMGCGCCCGCGGCCGCGG
CCGGCCCGCCKGCGCCGCAGGGCGAGGAGCTGTCGCCTAAGCGCGTCGTCTGCAGCCCCGACCTGCCCGTCTT
CTCGCTGACGCGCGCCTTCGAGGAGGCGGGCAAGGCGGCYGAGCAGGCGGCAGCCGTCGCCGCCGCCTCCG
CCACCGCCGCCGCCGCCGCCCCCGCRCAGGGCCCGCCCGGCGCCTTCCAGTGCTCCTTCTGCCAGAAGACGTTC
TCGCAGAAGAACACCTACCAGAACCAYGTGCGCTCGCACGGCAAGGAGGGCGACGACCCCTACCCGTGCACC
ATCTGCGGCAAGACGTTCGCGGTGCCGGCGCGCCTCACGCGCCACTTCCGCACGCACACGGGCGAGAAGCCG
TACCAGTGCGAGTTCTGCAAGAAGGCCTTCTCCGTCAAGGAGAACCTGAGCGTGCACCGGCGCATCCACACCA
AGGAGCGGCCGTACAAGTGCGACGTGTGCGAGCGCGCCTTCGAGCACAGCGGCAAGCTGCACCGGCACATG
CGCATCCACACGGGCGAGCGGCCGCACAAGTGCCAGGTGTGCTCCAAGACGTTCATCCAGAGCGGCCAGCTC
GTCATCCACATGCGCACGCACACGGGCGAGAAGCCGTACGTGTGCAAGGCGTGCGGCAAGGGCTTCACCTGC
TCCAAGCAGCTCAAGGTGCACACGCGCACGCACACGGGCGAGAAGCCCTACTCGTGCGACATCTGCGGCAAG
TCGTTCGGCTACAACCACGTGCTCAAGCTGCACCAGGTGGCGCACTACGGCGAGAAGGTGTACAAGTGCACC
ATCTGCAGCGAGACGTTCACCTCCAAGAAGACGATGGAGGTGCACATCAAGAGCCACTCGGAGCAGCAGCAC
CCCCAGCAACAGCAGCAGCAGGCGCCGCCTCAGCAGCCGCCGGCGGGGAGCTCTGCCCAGCAACCTTGGCAC
TCGCCATGGTCGCAACAGCAGCAACAGCAGCATCATCACCAGCAGCCGCAGCATCAACAACCGCCCCAGCAGC
AGCAACAGCTCGTCGTCCGAGGGCGTGGGCGGCAGCCTGACGCCGCCCGCCAGCGTCGAGGACGACGAGCA
CTTCGCGCGCGCCGACACGGCCGTGCCGCTGAACGAGCCGCCCGTCTACCTGTTCCCCGGGCCTCTGGGTTCG
ATGCGCGGTCGCGCCACGCCCTACGACCTGCCCGTACCCGCGTGCCTCGCGGCGTCCAGCCCCTCGCCGCCCCT
GACGCCGCCCTCCAGCAACCCCGTGTCGCACGTGCCCGCCGCCCCCGCGTCGCCCGACCGCCGCGACGCCAGC
TCGCTGCCGCCCCGCAAGCGCTGCAAGGAGATTCTCAAGTCCATCGCGGCCGCCGCCGCCGCCGAACCCGCCA
TCCGCTACAGCTCCGTCATACACTACGCGGGCGCCTCGTAAGGGCACCTCGCGAGCAGGACACTGGGACTTCT
CACGAGTGA

Figure S2. *LmKr-h1* sequence with primer sets indicated. Complete coding sequence of *L. migratoria Krüppel-homolog 1* mRNA as found on NCBI (accession number KJ425482.1) with localisation of the different primers that were selected for production of dsRNA constructs or for performing qRT-PCR assays. Sequence regions highlighted in yellow and green indicate the position of the primer sets that were used for synthesising the *dsLmKr-h1* and *dsLmKr-h1_2* constructs, respectively. Sequence regions highlighted in purple indicate the primer set used for qRT-PCR.

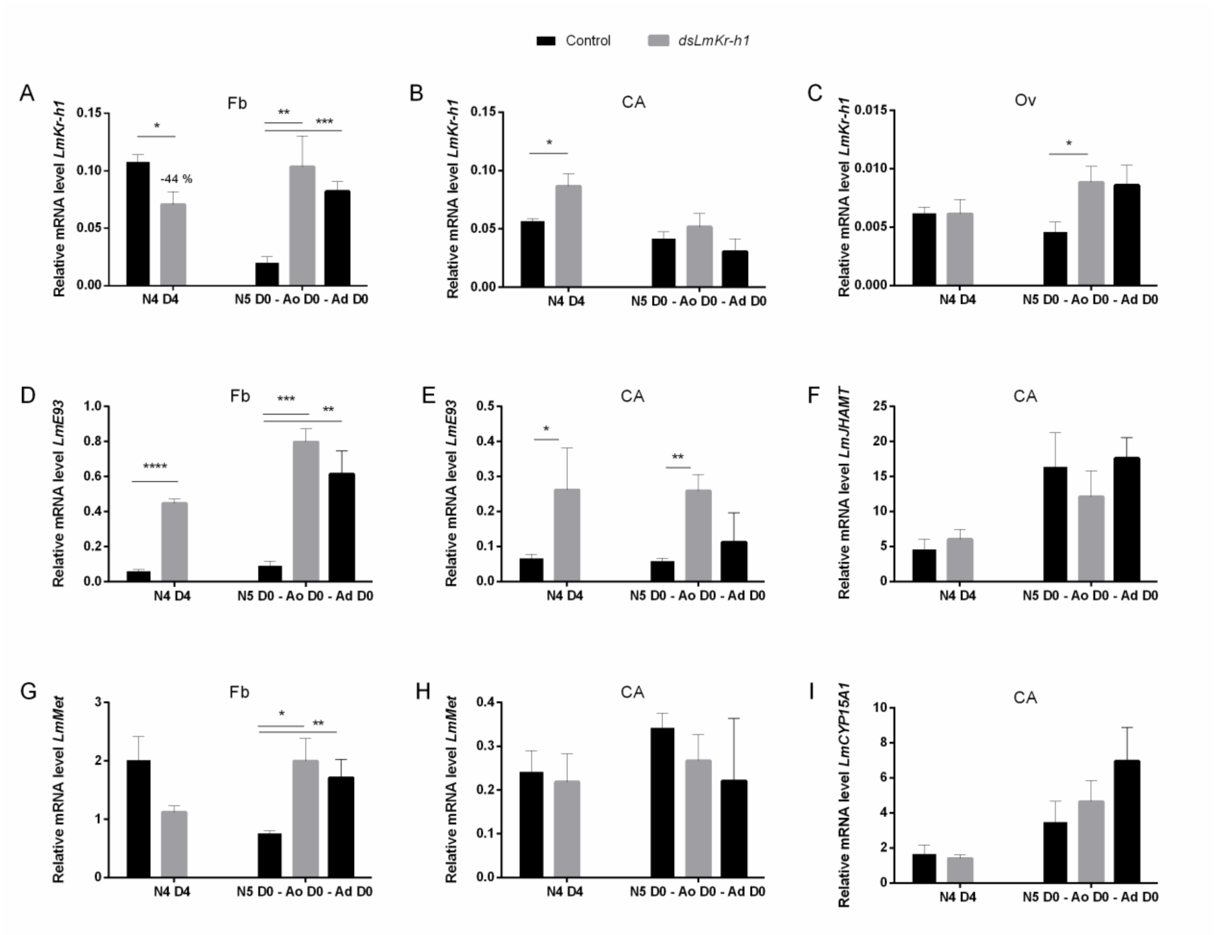

Figure S3. Relative transcript levels of MEKRE93 and JH biosynthesis pathway components in *dsLmKr-h1* or *dsGFP* injected female locusts. Relative *LmKr-h1* (**A** + **B**), *LmE93* (**D** + **E**) and *LmMet* (**G** + **H**) transcript levels were measured in the fat body (**A** + **D** + **G**) and the CA (**B** + **E** + **H**) of *dsLmKr-h1* (grey bars) and *dsGFP* (Control: black bars) injected 4-day-old fourth nymphal locusts (N4 D4), as well as in freshly moulted fifth nymphal (N5 D0), adultoid (Ao D0) and adult (Ad D0) female locusts. Relative *LmKr-h1* levels were also measured in the ovaries (**C**). Relative quantities of *LmJHAMT* (**F**) and *LmCYP15A1* (**I**) transcripts were measured in the CA. The data represent mean ± S.E.M. of four independent pools of three animals, run in duplicate and normalized to *rps13* + *CG13220*, *CG13220* + *TubA1* and *rp49* + *rps13* transcript levels for fat body, ovary and CA samples, respectively. Statistically significant differences between the measurements were found via a t-test on log-transformed data (with or without two-sided Welch's correction) and are indicated by (an) asterisk(s) (* $p < 0.05$; ** $p < 0.01$; *** $p < 0.001$; **** $p < 0.0001$).

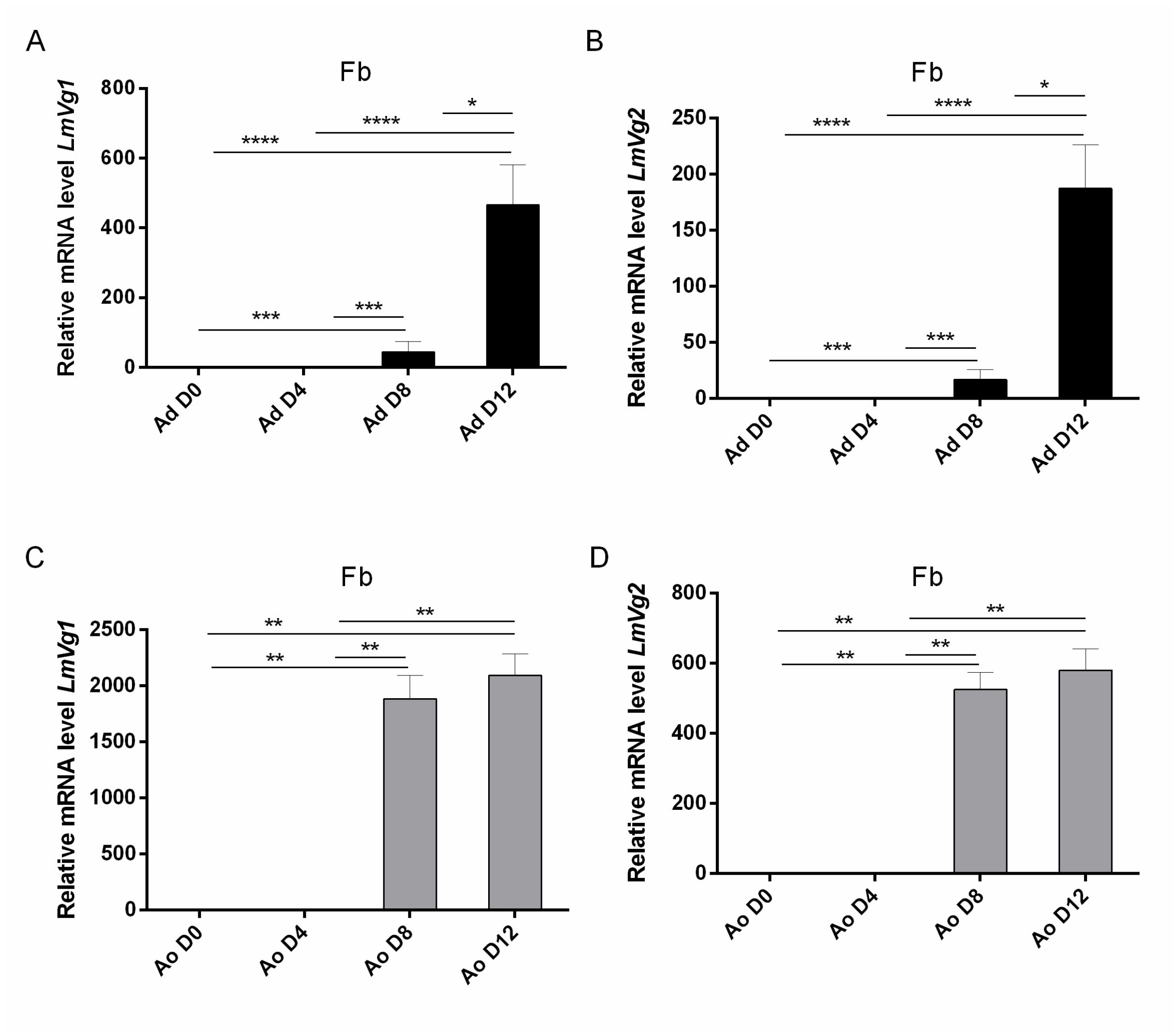

Figure S4. Relative *LmVg1* and *LmVg2* transcript levels of adult (*dsGFP* control) and adultoid (*dsLmKr-h1*) female locusts during the first gonadotrophic cycle. Relative transcript levels of *LmVg1* (**A** + **C**) and *LmVg2* (**B** + **D**) in the fat body of freshly moulted (D0), 4-day-old (D4), 8-day-old (D8) and 12-day-old (D12) adult (Ad: **A** + **B**) and adultoid (Ao: **C** + **D**) female locusts. The data represent mean ± S.E.M. of four independent pools of three animals, run in duplicate and normalized to *rps13* and *CG13220* transcript levels. Statistically significant differences between successive time points were found via a t-test on log-transformed data (with or without two-sided Welch's correction) and are indicated (an) asterisk(s) (* $p < 0.05$; ** $p < 0.01$; *** $p < 0.001$; **** $p < 0.0001$).

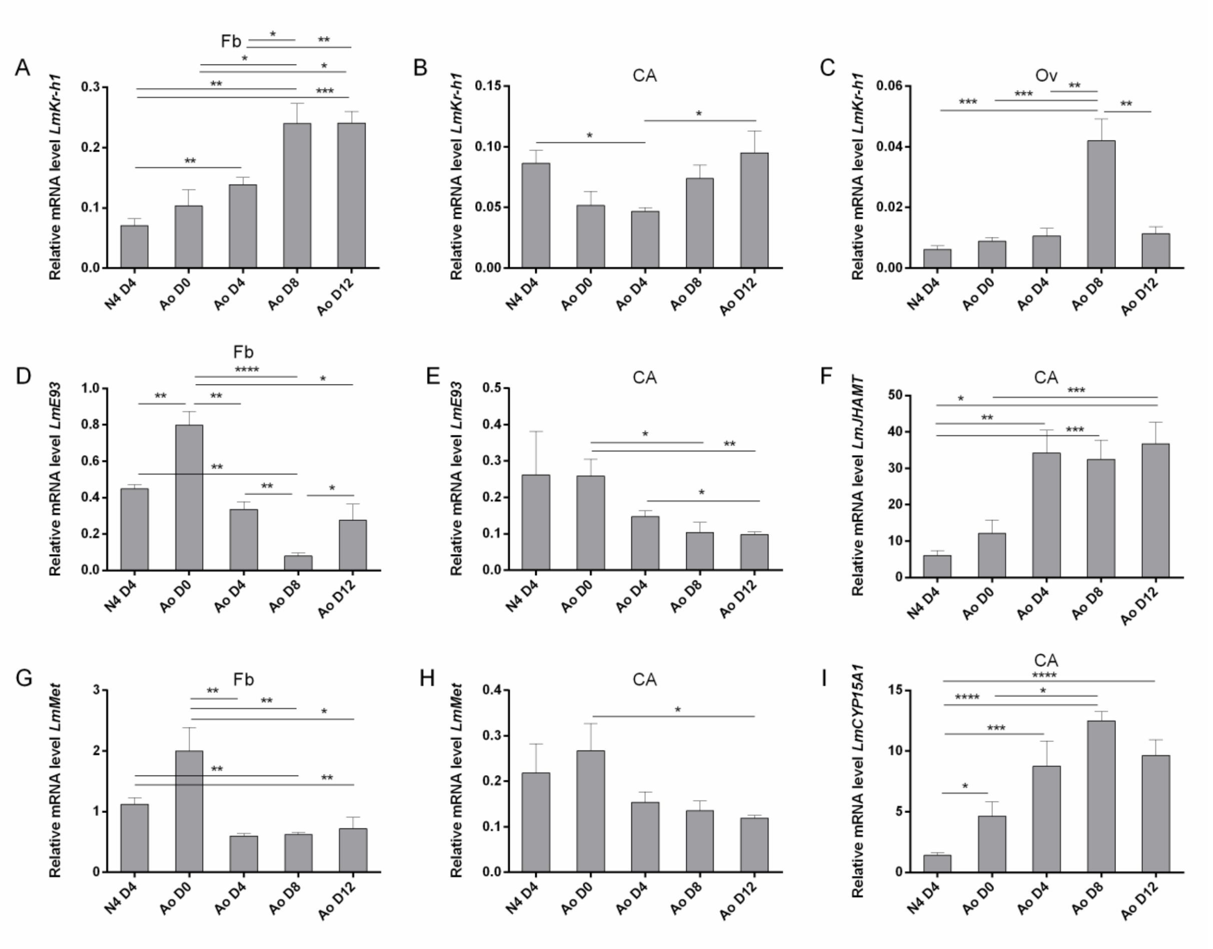

Figure S5. Relative transcript levels of MEKRE93 and JH biosynthesis pathway components in experimental (*dsLmKr-h1*) female locusts during the fourth nymphal stage and the first gonadotrophic cycle. Relative levels of *LmKr-h1* (**A** + **B**), *LmE93* (**D** + **E**) and *LmMet* (**G** + **H**) transcripts were determined in the fat body (**A** + **D** + **G**) and the CA (**B** + **E** + **H**) of experimental (*dsLmKr-h1*) females starting from day 4 in the fourth nymphal stage (N4 D4) until day 12 in the adultoid stage (Ao D12). Relative *LmKr-h1* levels were also measured in the ovaries (**C**). Relative quantities of *LmJHAMT* (**F**) and *LmCYP15A1* (**I**) transcripts were measured in the CA. The data represent mean ± S.E.M. of four independent pools of three animals, run in duplicate and normalized to *rps13* + *CG13220*, *CG13220* + *TubA1* and *rp49* + *rps13* transcript levels for fat body, ovary and CA samples, respectively. Statistically significant differences between the measurements were found via a t-test on log-transformed data (with or without two-sided Welch's correction) and are indicated by (an) asterisk(s) (* $p < 0.05$; ** $p < 0.01$; *** $p < 0.001$; **** $p < 0.0001$).

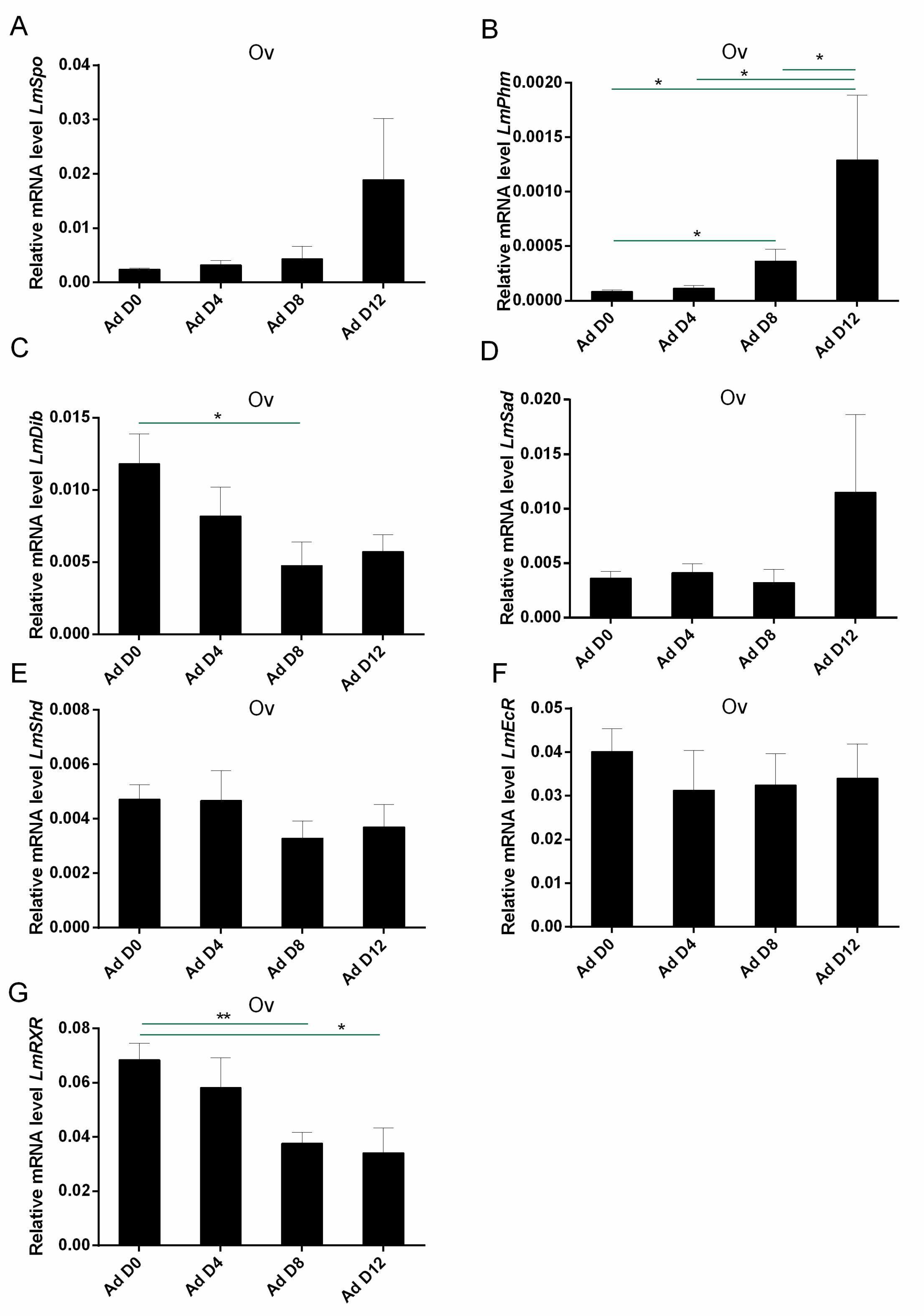

Figure S6. Relative transcript levels of several *Halloween* genes, as well as ecdysone receptor components *LmEcR and LmRXR,* during the first gonadotrophic cycle of adult (*dsGFP* control) female locusts. The relative quantities of *LmSpo* (**A**), *LmPhm* (**B**), *LmDib* (**C**), *LmSad* (**D**), *LmShd* (**E**), *LmEcR* (**F**) and *LmRXR* (**G**) transcripts were determined in the ovaries of control (*dsGFP*) adult females at 0 (Ad D0), 4 (Ad D4), 8 (Ad D8) and 12 (Ad D12) days after the final moult. The data represent mean ± S.E.M. of four independent pools of three animals, run in duplicate and normalized to *CG13220* and *TubA1* transcript

levels. Statistically significant differences between the measurements were found via a t-test on log-transformed data (with or without two-sided Welch's correction) and are indicated by (an) asterisk(s) (* $p < 0.05$; ** $p < 0.01$).

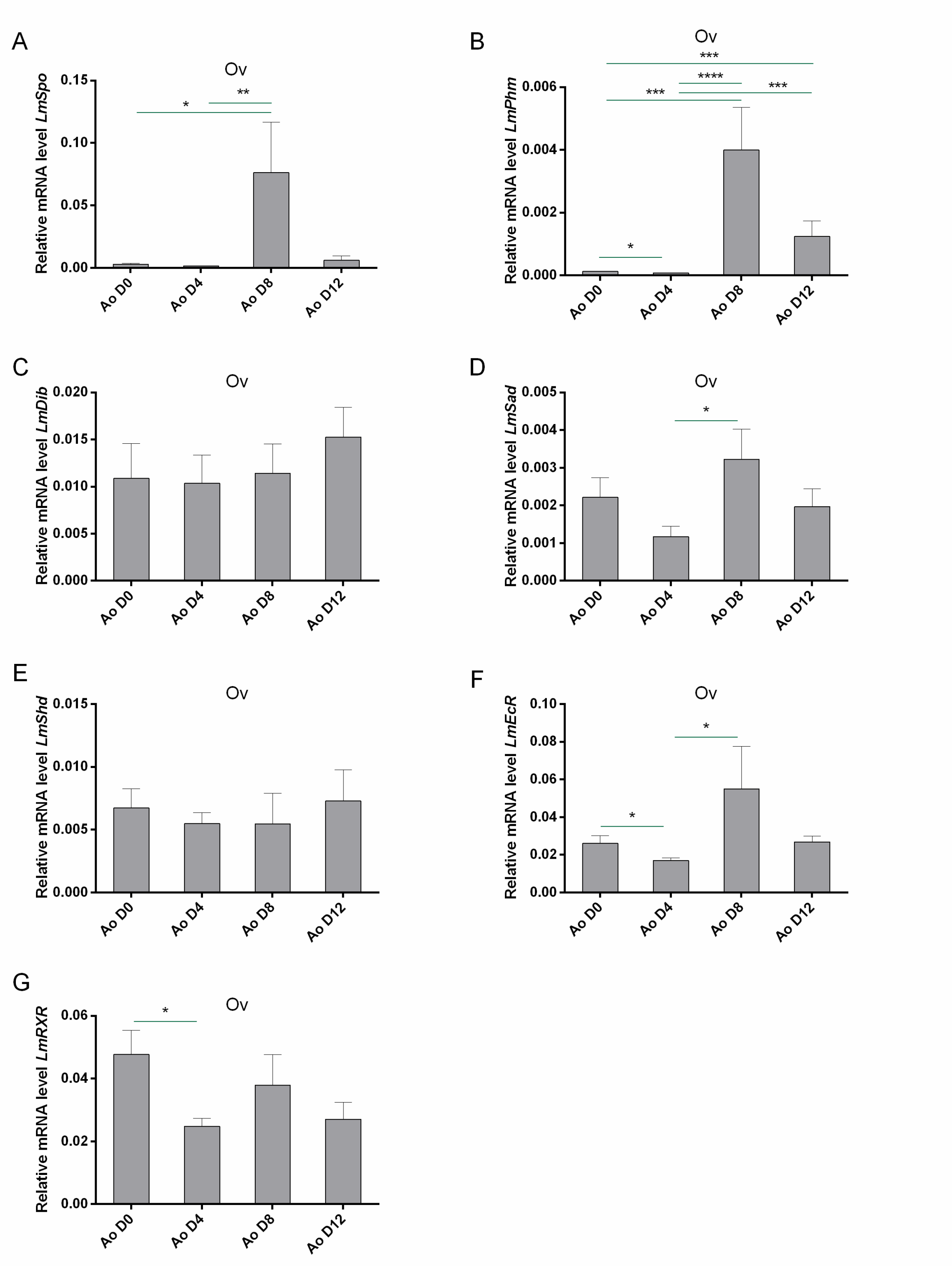

Figure S7. Relative transcript levels of several *Halloween* genes, as well as ecdysone receptor components *LmEcR and LmRXR,* during the first gonadotrophic cycle of adultoid (*dsLmKr-h1*) female locusts. The

relative quantities of *LmSpo* (**A**), *LmPhm* (**B**), *LmDib* (**C**), *LmSad* (**D**), *LmShd* (**E**), *LmEcR* (**F**) and *LmRXR* (**G**) transcripts were determined in the ovaries of adultoid (*dsLmKr-h1*) females at 0 (Ao D0), 4 (Ao D4), 8 (Ao D8) and 12 (Ao D12) days after the final moult. The data represent mean ± S.E.M. of four independent pools of three animals, run in duplicate and normalized to *CG13220* and *TubA1* transcript levels. Statistically significant differences between measurements were found via a t-test on log-transformed data (with or without two-sided Welch's correction) and are indicated by (an) asterisk(s) (* $p < 0.05$; ** $p < 0.01$; *** $p < 0.001$; **** $p < 0.0001$).

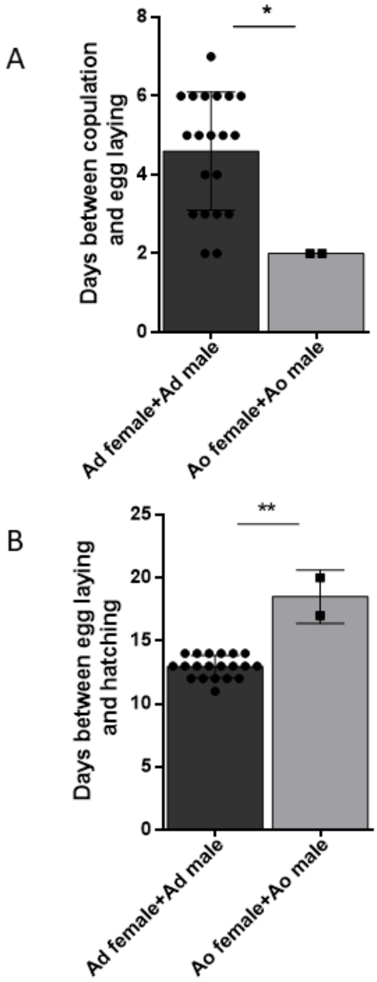

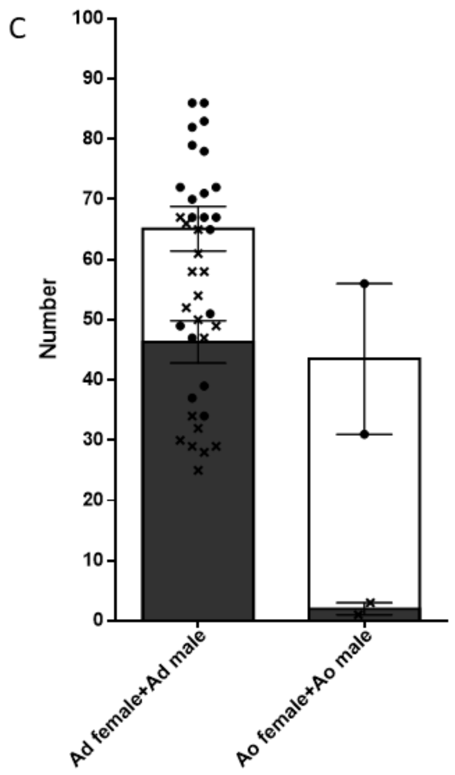

	Laid eggs (mean)	Hatchlings (mean)	Percentage hatchlings (mean)
Control	68.5	49.5	81.5
*dsKr-h1*_Ao	43.5	2.0	7.5
P value	ns	*	*

Figure S8. Time between mating and oviposition; incubation period between egg deposition and hatching; fecundity and fertility of adult (*dsGFP* control) and adultoid (*dsLmKr-h1*) *L. migratoria* females. Mating and oviposition were observed for 20 adult (Ad) (each combined with an adult male: 'Ad female + Ad male') and 14 adultoid (Ao) (each combined with an adultoid male: 'Ao female + Ao male') females starting on day 5 of their adult (Ad) or adultoid (Ao) stage. The periods (in number of days) between mating and oviposition (**A**), as well as between egg laying and hatching (**B**), were observed. The data represent mean ± S.E.M. for 20 adult ('Ad female + Ad male') and 2 adultoid ('Ao female + Ao male') females. Statistically significant differences (p) between the two conditions were found via a nonparametric Mann-Whitney test and are indicated by (an) asterisk(s) (* $p < 0.05$; ** $p < 0.01$). (**C**) The number of eggs per egg pod was counted, as well as the number of hatchlings. The data represent mean ± S.E.M. for 20 adult ('Ad female + Ad male') and 2 adultoid ('Ao female + Ao male') females, as well as

the individual number of eggs (o) or hatchlings (x) per egg pod. Statistically significant differences (p) between the two conditions were found via a nonparametric Mann-Whitney test and are indicated by an asterisk (* $p < 0.05$).

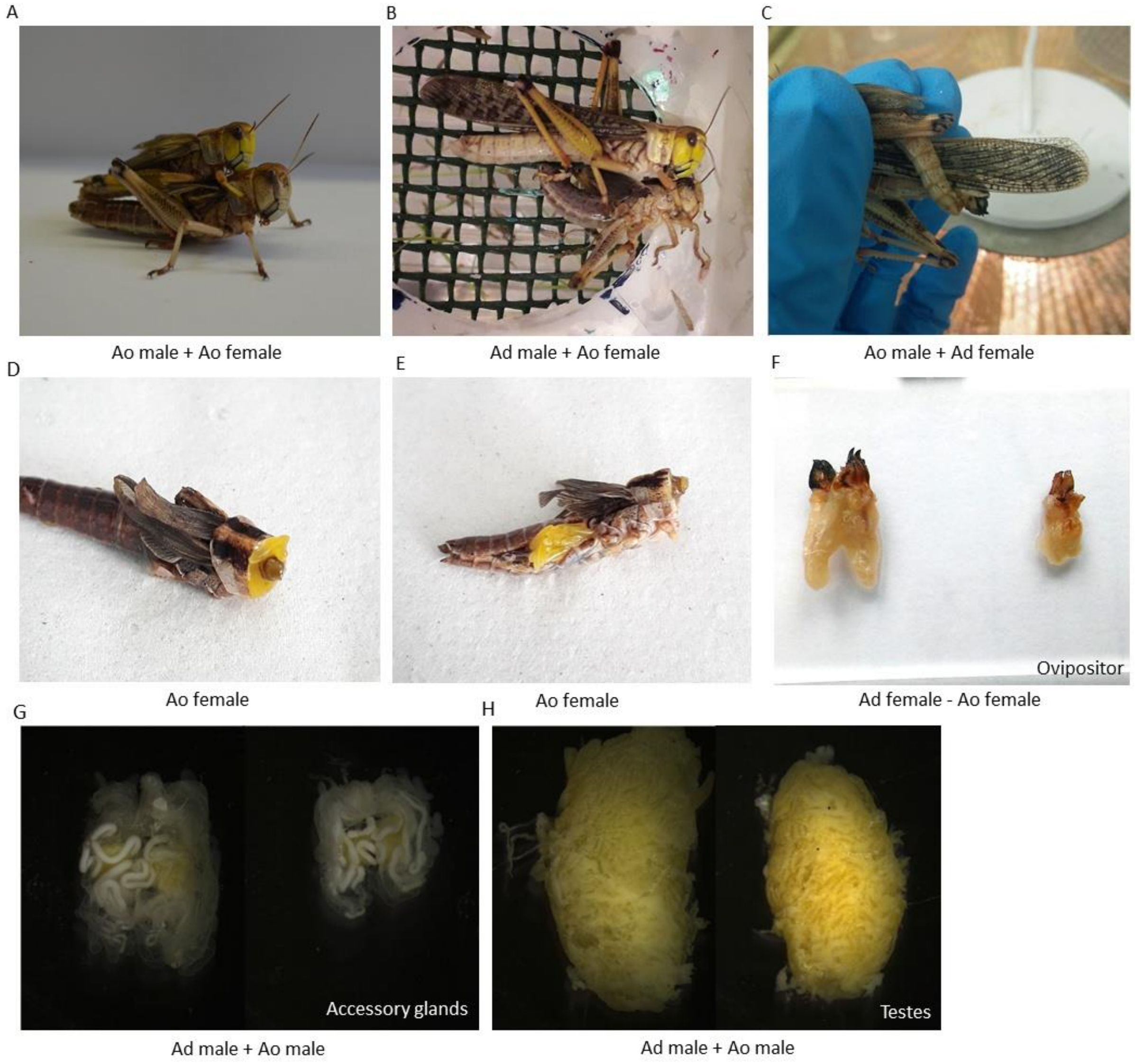

Figure S9. Mating and fecundity of adult (*dsGFP*) and adultoid (*dsKr-h1*) *L. migratoria*. The intention to mate as well as the actual connection between male and female genitalia were visually observed between adultoid (Ao) males and adultoid females (**A**), adult (Ad) control males and adultoid females (**B**), and adultoid males and adult control females (**C**). Adultoid females that were not successful in depositing their eggs were dissected. After decapitation (**D**) and making an incision in the abdomen (**E**), fully developed eggs were readily observed. (**F**) Dissected ovipositors from a control adult female (left) and an adultoid female (right). Dissected accessory glands (**G**) and testes (**H**) from a control adult male (left) and an adultoid male (right).

Table S1. Oligonucleotide sequences of primers used for qRT-PCR.

Reference/ Target Genes	Forward Primer	Reverse Primer
LmCG13220	5'- CGGCTTGGGAGTAAGGATGTT -3'	5'- CAATTAACGTGGACAGGAGCAA -3'
LmTubA1	5'- GTTGGCGTTTCGATTTTTCTTC -3'	5'- TGAGATACATTCACGCATTTTGC -3'
LmRPS13	5'- CGTGAAGGGTGATATTTTGCA -3'	5'- GCTGACTGGGATATACCCTTACCAG -3'
LmRP49	5'- CGCTACAAGAAGCTTAAGAGATCATG -3'	5'- CTTAAACCTACGGCGAACTCTGTT -3'
LmKr-h1	5'- GCCCTACTCGTGCGACAT -3'	5'- AGCTTGAGCACGTGGTTGTA -3'
LmMet	5'- GGTGCCTGAAGAAGAAGAAC -3'	5'- ATGGAGGTGATGAAGGAGAGAG -3'
LmE93	5'- CGCAAGTAGAGCAAGAACCT -3'	5'- TGGTCTTAGGAACATCCCAC-3'
LmVg1	5'- CCACTCAACGTCACCGCAGT -3'	5'- ACTTGAGCCAAATGGATGAG -3'
LmVg2	5'- ACGCCGACAGTGTTGGTATT -3'	5'- TGCTTCTCCGCAAAGACGTA -3'
LmCYP15A1	5'- GTCTGGGAGAAGGTCTTGCA -3'	5'- CTGGCACACTGATGCTGAAT -3'
LmJHAMT	5'- GTTCCTCGAGGACTGCATGA -3'	5'- CGTTTCCGCTCTTGTTGTTGT -3'
LmSpo	5'- CAACATCTTCACCTCCTACATGTG -3'	5'- GGGTCGTCGTAGTCGAAGGA -3'
LmPhm	5'- GGATGACGACGGCAACCT -3'	5'- TGCCTGTCTGGAAGGGTATG -3'
LmShd	5'- GTGCTCCAGTGACCAGTGAA -3'	5'- TTCGGGCAAGACAGATAGCA -3'
LmDib	5'- GCTGCGATCGTGCTACTTTG -3'	5'- GGATAGCCTGGCAACTTCTCA -3'
LmSad	5'- TCCTCGCCCTCCATGAAGA -3'	5'- CAACCAGGTCTACGGCTACA -3'
LmEcR	5'- AGCGCCCATCTCTTGTTGAA -3'	5'- CGCCTGTTGTCCACATATGC -3'
LmRXR	5'- AATGCCTTGCTATGGGAATG -3'	5'- TCCTTTGTTCGCTGCCTTTC -3'

Abbreviations: *Lm = Locusta migratoria*; *TubA1 = Tubulin A1*; *RPS13 = Ribosomal protein S13*; *RP49 = Ribosomal protein 49*; *Kr-h1 = Krüppel-homolog 1*; *Met = Methoprene-tolerant*; *E93 = Ecdysone-induced protein 93; Vg = Vitellogenin; CYP15A1 =* Cytochrome *P450 enzyme 15A1 (methyl farnesoate epoxidase); JHAMT = Juvenile hormone acid methyltransferase; Spo = Spook; Phm = Phantom; Shd = Shade; Dib = Disembodied; Sad = Shadow; EcR = Ecdysone receptor; RXR = Retinoid X receptor.*

Table S2. Oligonucleotide sequences of primers used for dsRNA synthesis. Oligonucleotide sequences indicated in 'bold' represent the T7 promoter sequences used for the preparation of dsRNA constructs.

dsRNA constructs	Forward primer	Reverse primer
LmKr-h1	5'- **TAATACGACTCACTATAGGGAGA** GAGAACCTGAGCGTGCACC -3'	5'- **TAATACGACTCACTATAGGGAGA** TGTGCACCTTGAGCTGCTT-3'
LmKr-h1_2	5'- **TAATACGACTCACTATAGGGAGA** ATGGTGGGCTACTTCAACGG-3'	5'- **TAATACGACTCACTATAGGGAGA** CCGCTCCTTGGTGTGGATG-3'
GFP	5'-**TAATACGACTCACTATAGGGAGA** AAGGTGATGCTACATACGGAA -3'	5'-**TAATACGACTCACTATAGGGAGA** ATCCCAGCAGCAGTTACAAAC-3'

Abbreviations: *Lm = Locusta migratoria; Kr-h1 = Krüppel-homolog 1; GFP = Green fluorescent protein.*
